# Data to establish Landé factors for 537.15 and 510.76 nm Fe I lines, and for rotational levels of the e ^6^Π and a ^6^Δ states of FeH

**DOI:** 10.1016/j.dib.2026.112687

**Published:** 2026-03-23

**Authors:** Amanda J. Ross, Patrick Crozet, Allan G. Adam, Timothy E. Blackmore, Dennis W. Tokaryk

**Affiliations:** a Institute Lumière Matière, Lyon 1 Université Claude Bernard & CNRS (UMR 5306), 10 rue Ada Byron, Campus Lyon-Tech La Doua, 69622 Villeurbanne, France; b Department of Chemistry, University of New Brunswick, 30 Dineen Drive, E3B 5A3 Fredericton, New Brunswick, Canada; c Department of Physics, University of New Brunswick, 8 Bailey Drive, E3B 5A3 Fredericton, New Brunswick, Canada

**Keywords:** Fe (I) Landé factors, Zeeman patterns in FeH, Excitation spectroscopy, Green system of iron monohydride

## Abstract

We present Zeeman spectra of the green e ^6^Π - a ^6^Δ origin band of the FeH molecule recorded in a low-pressure flame in magnetic fields up to 7000 Gauss. Zeeman splittings are well-resolved for the lowest rotational levels, and show sharp dependence on parity for a given rotational level. Magnetic field calibration was performed in situ, using Fe(I) transitions with strong magnetic response at 537.15 nm and 510.76 nm. The atomic spectra lead to revised Landé factors for the Fe (I) a ^3^F_3_ level at 12,560.934 cm^−1^ (*g* = 1.0873(2)), the z ^5^D_2_° level at 26,339.696 cm^−1^ (*g* = 1.49752(5)) and for the z ^3^F_2_° level at 32,133.991 cm^−1^ (*g* = 0.6806(3))*.*

## Graphical abstract

**Figure:**
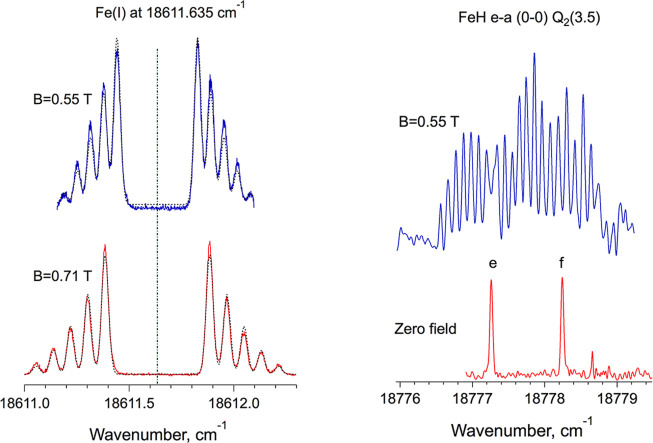

Specifications Table

**Table:** 

Subject	Atomic physics; Molecular physics
Specific subject area	Determination of Landé factors for 537.15 nm (18,611.635 cm^−1^) and 510.76 nm (19,573.056 cm^−1^) lines of atomic iron, and for rotational levels in the lowest vibrational levels of the e ^6^Π and a ^6^Δ excited electronic states of FeH*.*
Type of data	Figures of processed data (wavenumber calibrated) (.png files) Figure illustrating the experimental setup (.png) Tables with processed wavenumber and excitation spectra using wavelength-selected detection for molecular Zeeman data. ASCII (txt) format. Raw data for Fe(I) lines recorded at several magnetic field strengths (ASCII) Table of output from least-squares analysis (ASCII (txt) format)
Data collection	A flow of iron pentacarbonyl mixed with excited argon and hydrogen atoms produced Fe atoms and FeH in a primary vacuum environment. Excitation spectra were recorded at Doppler resolution, with a tuneable single-mode dye laser, calibrated against molecular iodine. A 0.75 m monochromator allowed wavelength-selective detection of Fe or FeH fluorescence, avoiding laser scatter, using a cooled photomultiplier as a detector. Fe or FeH signals were recorded simultaneously with reference fringes and I_2_ fluorescence using in-house software developed with the Igor Pro (Wavemetrics, Lake Oswego, OR, USA) package*.*
Data source location	Data collected at Department of Physics, University of New Brunswick, Canada. Stored at UNB, with copy at Institut Lumière Matière, Université Lyon 1, France*.*
Data accessibility	Repository name: Mendeley Data Data identification number: DOI: 10.17632/m4hgtk4f7d.1 Direct URL to data: https://data.mendeley.com/datasets/m4hgtk4f7d/1
Related research article	Landé factors for selected levels of the e ^6^Π and a ^6^∆ states of FeH, co-submitted to Journal of Molecular Spectroscopy [1].

## 1 Value of the Data

• The data provide the raw spectral observations from Zeeman studies of Fe and FeH transitions in the green region of the optical spectrum.
• The atomic Zeeman patterns for the a ^5^F_3_ − *z* ^5^D°_2_ transition of Fe(I) at 18,611.636 cm^−1^ lead to an improved value for the Landé factor of the atomic Fe(I) z ^5^D° level (*E* = 26,339.696 cm^−1^, configuration 3d^6^(^5^D)4s4p(^3^P°) *J* = 2), *g*=1.49752(5). Patterns recorded for the a ^3^F_3_ - z ^3^F°_2_ transition at 19,573.057 cm^−1^ give improved Landé factors for the level a ^3^F_3_ (*E* = 12,560.934 cm^−1^, configuration 3d^7^(^4^F)4 s *J* = 3), *g*=1.0873(2) and for level z ^3^F°_2_ (*E* = 32,133.991 cm^−1^, configuration 3d^6^(^5^D)4s4p(^3^P°) *J* = 2), *g*=0.6806(3). These provide useful values for magnetic field diagnostics.
• The molecular data reveal some unusual patterns in relative intensities that are not yet fully explained, and may be helpful for future studies.

The spectral data described here are expected to be useful in two contexts. One concerns projects constructing internationally recognized atomic and molecular data bases, in which magnetic responses are included, such as the NIST atomic data base http://physics.nist.gov/asd, or the EXOmol project, https://www.exomol.com, continually updating their compilation of data from multiple sources to provide the best possible simulations of spectra associated with stellar or planetary atmospheres. The files provided are particularly pertinent for interpreting future spectropolarimetric measurements, now possible at some ground-based telescopes. The other relates to the challenges for quantum chemistry, because Zeeman patterns and small perturbations seen in electronic spectra reveal details of configuration mixing, and thus provide reference points for ab initio studies, in addition to definition of field-free energy level information.

## 2 Background

The spectra presented here are part of a systematic investigation of the response of the iron monohydride radical to magnetic fields, because the FeH radical is seen in the atmospheres of cool (M dwarf) stars, and many molecular transitions are field-sensitive. The green bands investigated here were first identified in sun-spot spectra in 1972 [2], comparing with laboratory spectra recorded in a furnace at 2600 °C. We use lower temperatures with narrower Doppler widths to improve spectral resolution, and measure the distinctive profiles of resolved or partially-resolved molecular lines that can provide a diagnostic for magnetic field strength in remote environments. The strongest bands of FeH lie in the near infrared, but visible systems are of interest in this context too, because of increased detection sensitivity at shorter wavelengths. The low-lying electronic states of FeH are quartet or sextet multiplicity, and mixing occurs between them. As a result, the magnetic (Zeeman) response of FeH is difficult to predict as a function of molecular rotation for any given state, and needs to be measured in the laboratory. This study is the subject of a recent paper in J. Mol. Spectroscopy [1]. The individual line profiles result from the combination of upper- and lower-state effects. Since other systems are likely to share one of those investigated here, it is desirable to provide the raw information that can guide future work either in the lab, or from high-resolution telescopes.

## 3 Data Description

The data are available for download, https://data.mendeley.com/datasets/m4hgtk4f7d/1

Part 1 : Atomic data.

A compressed file Fe_atomic_line_data.zip contains 28 data files .txt in Ascii format for Zeeman spectra of the 537.15 nm (18,612 cm^−1^) line, and 8 measurements of the 510.76 nm (19,573 cm^−1^) line. The filenames indicate transition wavenumber in cm^−1^ and the date yymmdd the spectrum was taken. The accompanying ReadMe_Fe_atomic_line_data.txt file lists all of these, indicating magnetic field. If the spectra were NOT recorded with the laser beam in linear polarization, the selectivity in ΔM_J_ obtained with circular polarization conditions are noted as well.

The first column of each text file is the vacuum wavenumber (in cm^−1^) and the second column gives intensity (arbitrary units).

Part 2: Molecular data.

An Ascii file ReadMe_FeHfiles.txt describes the content of three compressed files Fn_Spectra.zip, where n refers to the spin component (F_1_ is the lowest in energy for both states). Each file contains excitation spectra in Ascii format for individual lines in the e ^6^Π- a ^6^Δ system of FeH. Within a given spin-component folder, the filename identifies the spectral transition (P, Q, R(J")), laser beam polarization and magnetic field strength. Each of these files has a matching thumbnail drawing, with extension .png.

The Ascii file SuppData_ZeemanFit_FeH_ea.txt provides the output from a linear least squares fit to term values and effective electronic Landé Factors in the e ^6^Π- a ^6^Δ system of FeH. The top of the file gives a list of fitted parameters, followed by assigned Zeeman components in F_1_-F_1_, F_2_-F_2_ and F_3_-F_3_ transitions.

The format for the parameter output is:

Level (e/a ^6^Λ, F_n_ TermEnergy (cm^−1^) 1σ StdDev (cm^−1^) *g*_elec_eff_ 1σ StdDev(*g*__elec_eff_)

Lower state term energies (fixed parameters) have been taken from Carter & Brown [3].

The second part of the file contains the list of peak positions retrieved from the spectra, that are to be used in analysis. The format for the spectral line data is:

Line-ID Wavenumber(cm^−1^) Est. Unc. (cm^−1^) M_J_" ΔM_J_ Magnetic flux (tesla) obs-calc (cm^−1^)

Zero-field wavenumbers have been taken from Goodridge et al. [4]*.*

## 4 Experimental Design, Materials and Methods

**Fig. 1:**
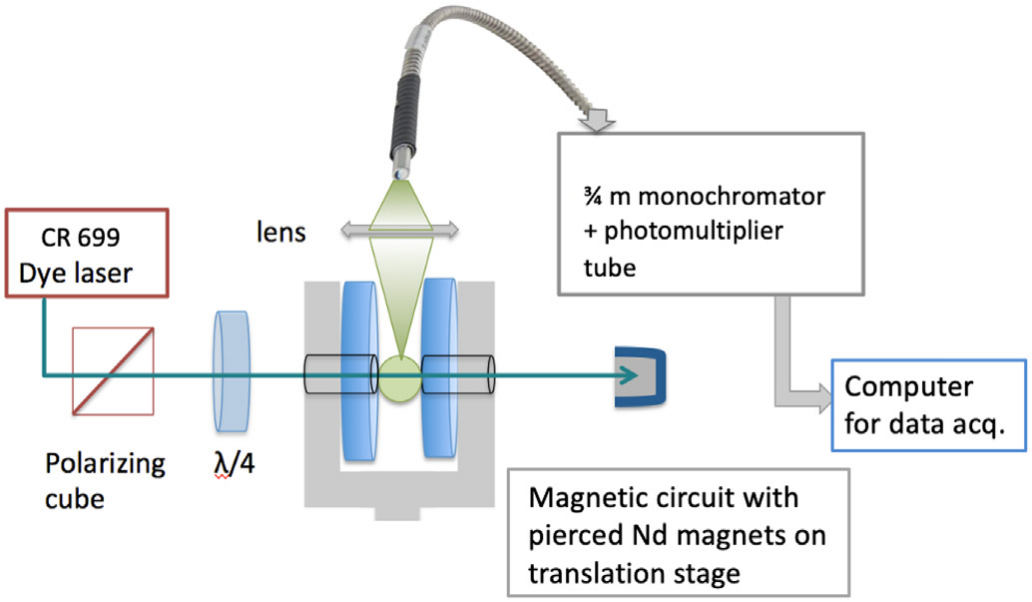
Schematic view of the experimental arrangement.

The experimental arrangement is illustrated in Fig. 1. We perform laser excitation on Fe atoms or FeH molecules produced by reacting H atoms with iron pentacarbonyl under vacuum. This method of production was suggested by Beaton et al. [5], and implemented at UNB by adapting a Broida-type oven to accommodate a very gentle flow of Fe(CO)_5_ into products of a flowing mixture of H_2_ and Ar excited with a microwave discharge[6]. The zero-field spectrum of the e ^6^Π-a ^6^Δ system of FeH has already been recorded with a similar set-up [4,7].

Output from a tuneable single-mode dye laser crosses the plasma, exciting atomic or molecular fluorescence. This is transferred to a selective detection assembly. The 0.75 m monochromator acts as an adjustable filter to eliminate unwanted contributions from laser scatter and from the discharge source used to produce Fe and H atoms. The molecular transitions were sometimes accidentally close to atomic lines; by detecting on a different branch (R/Q) or at higher order, background noise could be minimized by appropriate settings on the monochromator. A Peltier-cooled C31034 photomultiplier tube from RCA served as detector. Current from the photomultiplier was processed with a Keithley 414S picoammeter or a SR-830 lock-in amplifier using a mechanical chopper to modulate the laser at 1 kHz. For particularly weak lines, photon-counting was used instead: the photomultiplier signal was fed to an SR445A preamplifier and processed with a Stanford Research Systems SR400 photon counter. Signals were recorded simultaneously with Fabry-Perot fringes and molecular iodine absorption for calibration using in-house software developed with the IGOR-Pro package from Wavemetrics, Lake Oswego, OR, USA.

The key to this experiment was to record spectra from a small volume of plasma, around 1 mm^3^, where the magnetic field generated by annular NdFeB magnets could be considered uniform. This entailed using a collimated laser beam (diameter ∼ 1 mm through the plasma plume) aligned through the centres of the annular magnets, and collecting fluorescence from a short (∼1.5 mm) zone where laser beam and molecules interact, midway between the magnet stacks.

**Fig. 2:**
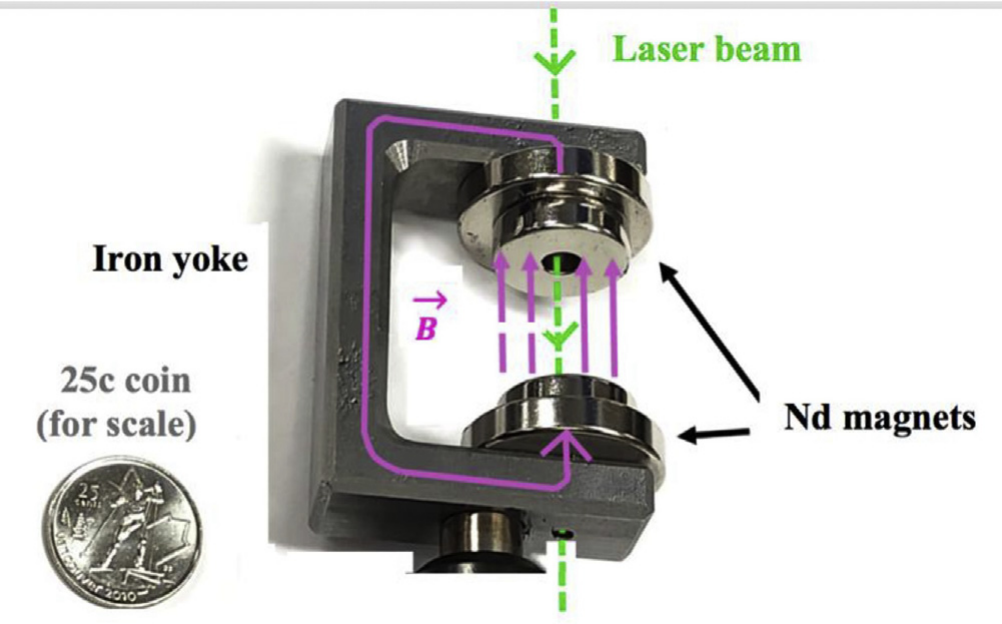
The magnetic circuit (photograph).

The magnetic circuit consists of sets of pierced magnets (typically 1″ in diameter with 1/8″ or 1/4″ central holes,) mounted on an iron yoke also drilled with 1/8″ holes, to allow the laser beam to cross the flux of molecules at 90°. Thicker magnets, leaving a smaller distance between them, produced stronger magnetic fields acting on the plasma generated at the centre of the circuit. Three different arrangements of magnets allowed us to work with magnetic flux values close to 0.46, 0.55 and 0.71 T. The assembly is shown in Fig. 2. It is similar to the arrangement used by Steimle et al. [7] to study the Zeeman effect in MnH, except that we send the laser beam through the hole in the magnets instead of a molecular beam.

Approximate magnetic flux values could be read on a Hall probe while the magnets were being mounted outside the vacuum chamber, but a true measurement is needed in situ, and varies slightly according to optimization of signal according to the flame conditions and the associated zone of brightest fluorescence along the path of the laser beam. Very slight changes in the alignment of the laser beam resulted in small variations in $\vec{B}$. This is not unexpected: Sargsyan et al. [8] mention field gradients of the order of 0.015 T/mm being a potential source of difficulty in their investigations of Paschen-Back behaviour in Rb atoms. The variations we encountered within a given magnet stack from day to day were smaller than this, possibly because irises were placed on the laser beam path to keep measurements as consistent as possible. In ref. [9], Sargsyan et al. advocate the use of nanometric cells to limit dimension of the atomic source to avoid such variations. Our approach is similar, selecting just the brightest part of the laser-induced fluorescence for transfer to detection optics. Nevertheless, each molecular measurement had to be accompanied by a flux-calibration measurement, with no changes in alignment made between the two. Provided that measurements are made within ± 2 mm of the centre of the magnet stack, we estimate that flux gradients are <0.004 T/mm in our experiments.

**Fig. 3:**
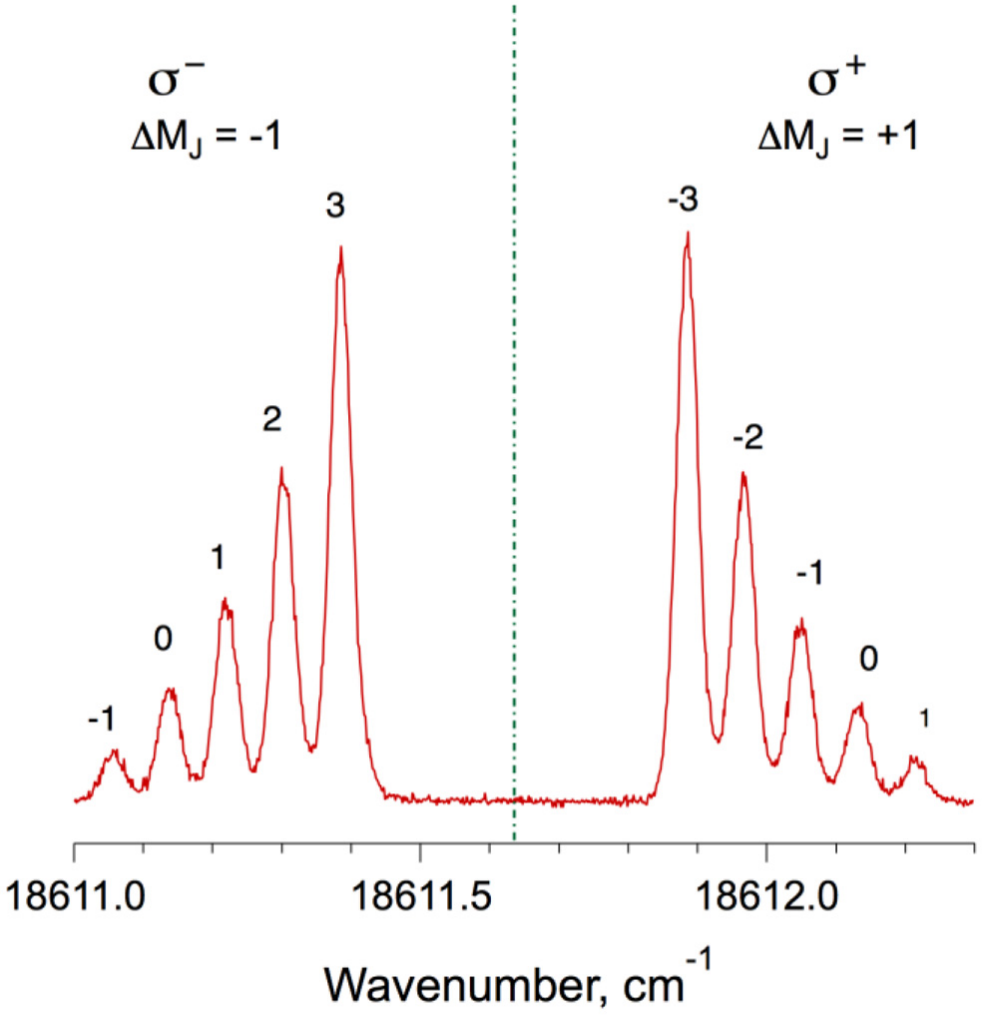
Zeeman structures in the Fe(I) line at 537.15 nm, recorded in linear polarisation, indicating lower-state M_J"_ quantum numbers. Recorded at $|\vec{B}|=$ 0.71 T.

The discharge source produces metastable argon and iron atoms, some of which are sensitive to magnetic field. Several iron lines were accessible with the laser dye needed to record the molecular spectrum, and some of them have easily-resolved Zeeman patterns. Landé factors for the selected transitions have been tabulated at the NIST Atomic Spectra Database [10], so either upper- or lower-state combination differences taken between the resolved M_J_ components of the Zeeman-split atomic line can be used to determine magnetic flux. We selected Fe(I) lines at 537.15 nm (18,611.636 cm^−1^) and at 510.76 nm (19,573.057 cm^−1^) for this purpose, and recorded their Zeeman spectra in laser excitation, recording Fabry-Perot fringes and molecular iodine absorption [11] simultaneously to provide a reliable wavenumber scale. The atomic line profiles are also an excellent indicator of inhomogeneity in $\vec{B}$. In the example shown in Fig. 3, the weak, outermost components are slightly broader than the stronger ones that are subject to smaller shifts. In a truly homogeneous field, all components would be expected to have Doppler-broadened profiles, with full-width at half-maximum (fwhm) ∼ 0.033 cm^−1^. The strong, narrow peaks in Fig. 3 have fwhm 0.035 cm^−1^, and the outermost, weak features have fwhm 0.038(3) cm^−1^. Both fits to peak position, and global profile fits allow us to define an average value of $|\vec{B}|$ to within 1% uncertainty. If the atomic spectrum exhibited broadening stronger than shown in Fig. 3, alignment was readjusted before proceeding with measurements.

We found that the literature values for the Landé factors for the upper and lower states of the Fe of the 537.15 nm (18,611.64 cm^−1^) line returned different magnetic field strengths, depending on whether upper-state or lower-state combination differences were analysed. Further investigation showed that while the lower state Landé factor of 1.24988(4) was of high quality [5], the value for the upper state (1.503) was suspect. The first step was therefore to refine the upper-state Landé factor for the 537.15 nm line, by recording the Zeeman-resolved patterns illustrated in Fig. 3 several times, and with different magnetic flux values. Upper-state combination differences were extracted from peak positions σ^+^(M_J"_) – σ^−^(M_J"_). These differences are directly proportional to magnetic flux $\vec{B}$ and the upper state Landé factor *g*'. Details of the analysis are given in the accompanying research paper [1]; the revised Landé factor for the z ^5^D_2_° level of Fe(I) at 26,339.696 cm^−1^ is 1.49752(5).

We also took a series of spectra for a second Fe(I) transition at 510.76 nm (19,573.056 cm^−1^), to generate effective Landé factors that can be used to calibrate magnetic flux in our experiments.

**Fig. 4:**
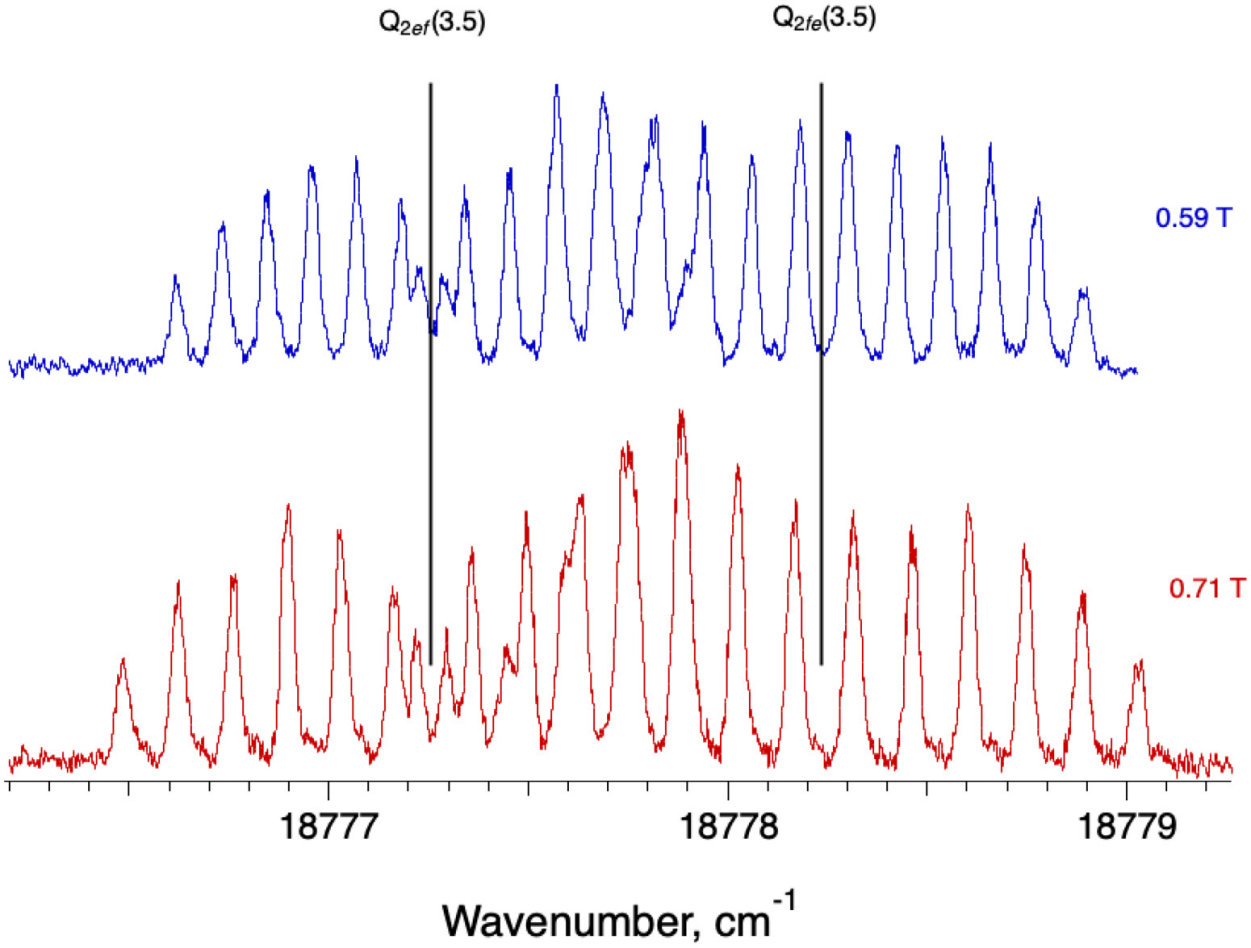
Zeeman structures in the Q_2_(3.5) 0–0 line in the e ^6^Π - a ^6^Δ FeH, recorded in linear polarisation at 0.59 and 0.71 T. Most of the 28 expected ΔM_J_ = ±1 components are resolved, with overlap between the contributions from the two parity components broadening and distorting some of the features in the central region. Vertical lines indicate the position of the Q_2_e_f_ (3.5) and Q_2fe_ (3.5) transitions in field-free conditions.

The molecular spectra of some FeH e ^6^Π - a ^6^∆ transitions are discussed in detail in ref. [1], with raw data supplied in ascii format on Mendeley Data. They are transitions between two excited states of FeH, the lower being the ground state of the sextet manifold, located 1891 cm^−1^ above the true X ^4^∆ ground state. The profiles are quite different from one spin-component to another; P branches are resolved only at very low J, Q-lines, on the other hand, spread over >1 cm^−1^. An example is shown in Fig. 4, illustrating the Zeeman structure of the first Q line of the second spin component. The zero-field line positions are known for the two parity components of this transition from ref. [4], and are indicated by solid vertical lines. The spacings between M_J_ components of a given parity component are constant, but differ between the two parity components. Although the difference is hardly perceptible in the figure, the spacing between consecutive peaks at the extreme right-hand side of Fig. 4 (ΔM_J_ = +1 for Q_2fe_ (3.5) is 5% larger than found between those at the extreme left-hand side, (ΔM_J_ = −1 for Q_2ef_ (3.5))*.* As in Fig 3, the weak, outermost components of the Zeeman patterns show evidence for small field inhomogeneity, with a fwhm for the lowest wavenumber peak (at 18,776.484 cm^−1^ in the lower trace) 0.04 cm^−1^ is nearly 10% broader than the 4^th^ peak from the left (fwhm 0.037 cm^−1^). A profile fit of this particular spectrum including field inhomogeneity as a free parameter returned $|\vec{B}|$ = 0.711 T and d$\vec{B}$ = 0.004 T/mm.

Remark: This paper is intended to make available the observed Doppler-limited profiles of the transitions in FeH we were able to record in laser excitation. They are short scans of individual lines, sometimes exhibiting unusual intensities, probably arising from mixing between neighbouring electronic states.

## Limitations

The spectra are Doppler-limited, so not all structures are fully resolved. Since the intensity of any given transition is spread amongst its M_J_ components, only the strongest lines could be studied. Transitions from higher rotational levels are readily excited in zero-field conditions, but could not be recorded with the signal/noise ratio achieved here.

## Ethics Statement

The authors have read and follow the ethical requirements for publication in Data in Brief and confirm that the current work does not involve human subjects, animal experiments, or any data collected from social media platforms*.*

## CRediT Author Statement

**Amanda J. Ross:** Investigation, Data Analysis, Data Curation, Writing – Original Draft Preparation, Review & Editing; **Patrick Crozet:** Methodology, Investigation, Software, Writing – Review & Editing; **Allan G. Adam:** Resources, Investigation, Writing – Review & Editing; **Timothy E. Blackmore:** Investigation, Writing – Review & Editing; **Dennis W. Tokaryk:** Resources, Investigation, Software, Data Analysis, Writing – Original Draft Preparation, Review & Editing*.*

## Data Availability

Mendeley DataZeeman spectra of the green e-a transition of FeH and of two Fe(l) lines (Original data).
